# 2-Eth­oxy­methyl-6-ethyl-2,3,4,5-tetra­hydro-1,2,4-triazine-3,5-dione

**DOI:** 10.1107/S1600536811055747

**Published:** 2012-01-11

**Authors:** Nasser R. El-Brollosy, Ali A. El-Emam, Omar A. Al-Deeb, Seik Weng Ng

**Affiliations:** a Department of Pharmaceutical Chemistry, College of Pharmacy, King Saud University, Riyadh 11451, Saudi Arabia; b Department of Chemistry, University of Malaya, 50603 Kuala Lumpur, Malaysia; c Chemistry Department, Faculty of Science, King Abdulaziz University, PO Box 80203 Jeddah, Saudi Arabia

## Abstract

The 1,2,4-triazine ring of the title compound, C_8_H_13_N_3_O_3_, is nearly planar (r.m.s. deviation = 0.019 Å). The imino group is hydrogen-bond donor to the exocyclic O atom at the 5-position of an adjacent mol­ecule, the N—H⋯O hydrogen bond generating a chain parallel to the *b* axis.

## Related literature

For the synthesis, see: El-Brollosy (2008 ▶).
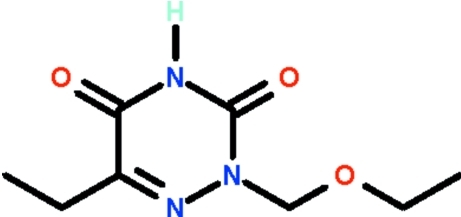


## Experimental

### 

#### Crystal data

C_8_H_13_N_3_O_3_
*M* *_r_* = 199.21
Monoclinic, 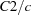

*a* = 20.4078 (19) Å
*b* = 4.4343 (3) Å
*c* = 22.6285 (17) Å
β = 111.813 (10)°
*V* = 1901.1 (3) Å^3^
*Z* = 8
Mo *K*α radiation
μ = 0.11 mm^−1^
*T* = 100 K
0.35 × 0.25 × 0.15 mm

#### Data collection

Agilent SuperNova Dual diffractometer with an Atlas detector
Absorption correction: multi-scan (*CrysAlis PRO*; Agilent, 2010 ▶) *T*_min_ = 0.963, *T*_max_ = 0.984
3623 measured reflections
2174 independent reflections
1739 reflections with *I* > 2σ(*I*)
*R*_int_ = 0.023

#### Refinement

*R*[*F* ^2^ > 2σ(*F* ^2^)] = 0.042
*wR*(*F* ^2^) = 0.107
*S* = 1.06
2174 reflections
131 parameters
H atoms treated by a mixture of independent and constrained refinement
Δρ_max_ = 0.31 e Å^−3^
Δρ_min_ = −0.28 e Å^−3^

### 

Data collection: *CrysAlis PRO* (Agilent, 2010 ▶); cell refinement: *CrysAlis PRO*; data reduction: *CrysAlis PRO*; program(s) used to solve structure: *SHELXS97* (Sheldrick, 2008 ▶); program(s) used to refine structure: *SHELXL97* (Sheldrick, 2008 ▶); molecular graphics: *X-SEED* (Barbour, 2001 ▶); software used to prepare material for publication: *publCIF* (Westrip, 2010 ▶).

## Supplementary Material

Crystal structure: contains datablock(s) global, I. DOI: 10.1107/S1600536811055747/xu5421sup1.cif

Structure factors: contains datablock(s) I. DOI: 10.1107/S1600536811055747/xu5421Isup2.hkl

Supplementary material file. DOI: 10.1107/S1600536811055747/xu5421Isup3.cml

Additional supplementary materials:  crystallographic information; 3D view; checkCIF report

## Figures and Tables

**Table 1:** Hydrogen-bond geometry (Å, °)

*D*—H⋯*A*	*D*—H	H⋯*A*	*D*⋯*A*	*D*—H⋯*A*
N1—H1⋯O1^i^	0.89 (2)	1.95 (2)	2.837 (2)	174.1 (17)

Symmetry code: (i) 
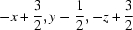
.

**Fig. 1.**
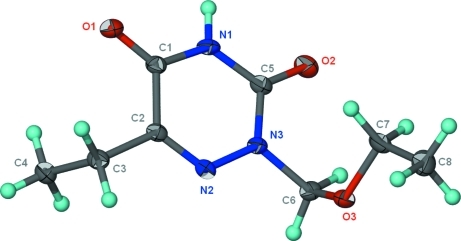
Thermal ellipsoid plot (Barbour, 2001) of C8H13N3O3 at the 70% probability level; hydrogen atoms are drawn as spheres of arbitrary radius.

**Fig. 2.**
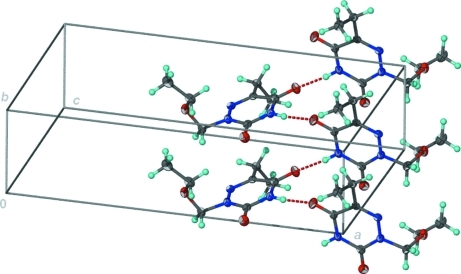
Hydrogen-bonded chain structure.

## supplementary crystallographic information

### Comment

The compound (Scheme I) was synthesized for an evaluation of its anti-microbial properties (El-Brollosy, 2008). The 1,2,4-triazine ring is planar; the C atom at the 6-position deviates by 0.155 (2) Å from the mean plane whereas the C atom at the 2-position deviates from the mean plane by 0.081 (2) Å in the opposite direction (Fig. 1). The amino group is hydrogen-bond donor to the exocyclic O atom at the 5-position, the hydrogen bond generating a linear chain parallel to the *b*-axis of the monoclinc unit cell (Table 1, Fig. 2).

### Experimental

The compound was synthesized by using a reported method (El-Brollosy, 2008), and was recrystallized from ethanol.

### Refinement

Carbon-bound H-atoms were placed in calculated positions [C–H 0.95 to 0.98 Å, *U*_iso_(H) 1.2 to 1.5*U*_eq_(C)] and were included in the refinement in the riding model approximation.

The imino H-atom was located in a difference Fourier map, and was freely refined.

The -2 0 2 reflection that was affected by the beam-stop was omitted.

### Crystal data

**Table:**

C_8_H_13_N_3_O_3_	*F*(000) = 848
*M**_r_* = 199.21	*D*_x_ = 1.392 Mg m^−^^3^
Monoclinic, *C*2/*c*	Mo *K*α radiation, λ = 0.71073 Å
Hall symbol: -C 2yc	Cell parameters from 1592 reflections
*a* = 20.4078 (19) Å	θ = 2.3–27.5°
*b* = 4.4343 (3) Å	µ = 0.11 mm^−^^1^
*c* = 22.6285 (17) Å	*T* = 100 K
β = 111.813 (10)°	Prism, colorless
*V* = 1901.1 (3) Å^3^	0.35 × 0.25 × 0.15 mm
*Z* = 8	

### Data collection

**Table:**

Agilent SuperNova Dual diffractometer with an Atlas detector	2174 independent reflections
Radiation source: SuperNova (Mo) X-ray Source	1739 reflections with *I* > 2σ(*I*)
Mirror	*R*_int_ = 0.023
Detector resolution: 10.4041 pixels mm^-1^	θ_max_ = 27.6°, θ_min_ = 3.4°
ω scan	*h* = −26→26
Absorption correction: multi-scan (CrysAlis PRO; Agilent, 2010)	*k* = −5→5
*T*_min_ = 0.963, *T*_max_ = 0.984	*l* = −15→29
3623 measured reflections	

### Refinement

**Table:**

Refinement on *F*^2^	Primary atom site location: structure-invariant direct methods
Least-squares matrix: full	Secondary atom site location: difference Fourier map
*R*[*F*^2^ > 2σ(*F*^2^)] = 0.042	Hydrogen site location: inferred from neighbouring sites
*wR*(*F*^2^) = 0.107	H atoms treated by a mixture of independent and constrained refinement
*S* = 1.06	*w* = 1/[σ^2^(*F*_o_^2^) + (0.0432*P*)^2^ + 1.2698*P*] where *P* = (*F*_o_^2^ + 2*F*_c_^2^)/3
2174 reflections	(Δ/σ)_max_ = 0.001
131 parameters	Δρ_max_ = 0.31 e Å^−^^3^
0 restraints	Δρ_min_ = −0.28 e Å^−^^3^

### Fractional atomic coordinates and isotropic or equivalent isotropic displacement parameters (Å^2^)

**Table:**

	*x*	*y*	*z*	*U*_iso_*/*U*_eq_	
O1	0.73492 (5)	0.9252 (3)	0.68461 (5)	0.0201 (3)	
O2	0.58065 (6)	0.2354 (3)	0.70746 (5)	0.0214 (3)	
O3	0.42473 (5)	0.6126 (2)	0.59416 (5)	0.0172 (3)	
N1	0.65902 (7)	0.5753 (3)	0.69639 (6)	0.0152 (3)	
N2	0.56241 (6)	0.7358 (3)	0.57988 (6)	0.0138 (3)	
N3	0.54722 (6)	0.5285 (3)	0.61778 (5)	0.0144 (3)	
C1	0.67786 (7)	0.7953 (3)	0.66355 (7)	0.0141 (3)	
C2	0.62375 (7)	0.8617 (3)	0.59987 (6)	0.0130 (3)	
C3	0.64247 (8)	1.0709 (3)	0.55670 (7)	0.0159 (3)	
H3A	0.6664	1.2510	0.5810	0.019*	
H3B	0.5987	1.1387	0.5221	0.019*	
C4	0.69101 (8)	0.9188 (4)	0.52750 (7)	0.0197 (3)	
H4A	0.7019	1.0617	0.4994	0.030*	
H4B	0.6672	0.7418	0.5029	0.030*	
H4C	0.7349	0.8559	0.5616	0.030*	
C5	0.59417 (8)	0.4319 (3)	0.67624 (7)	0.0152 (3)	
C6	0.47519 (8)	0.4069 (3)	0.59096 (7)	0.0167 (3)	
H6A	0.4642	0.3508	0.5459	0.020*	
H6B	0.4726	0.2216	0.6144	0.020*	
C7	0.42810 (8)	0.6726 (4)	0.65804 (7)	0.0200 (3)	
H7A	0.4709	0.7914	0.6818	0.024*	
H7B	0.4300	0.4809	0.6810	0.024*	
C8	0.36268 (9)	0.8478 (4)	0.65258 (8)	0.0252 (4)	
H8A	0.3635	0.8925	0.6953	0.038*	
H8B	0.3207	0.7277	0.6292	0.038*	
H8C	0.3614	1.0369	0.6298	0.038*	
H1	0.6913 (10)	0.515 (4)	0.7331 (9)	0.024 (5)*	

### Atomic displacement parameters (Å^2^)

**Table:**

	*U*^11^	*U*^22^	*U*^33^	*U*^12^	*U*^13^	*U*^23^
O1	0.0179 (6)	0.0261 (6)	0.0138 (5)	−0.0056 (5)	0.0028 (4)	−0.0018 (4)
O2	0.0237 (6)	0.0226 (6)	0.0166 (5)	−0.0020 (5)	0.0059 (4)	0.0063 (5)
O3	0.0153 (5)	0.0246 (6)	0.0112 (5)	0.0001 (5)	0.0043 (4)	−0.0008 (4)
N1	0.0148 (6)	0.0179 (7)	0.0095 (6)	0.0019 (5)	0.0007 (5)	0.0013 (5)
N2	0.0168 (6)	0.0133 (6)	0.0118 (6)	0.0010 (5)	0.0059 (5)	0.0008 (5)
N3	0.0141 (6)	0.0171 (6)	0.0102 (6)	−0.0017 (5)	0.0026 (5)	0.0019 (5)
C1	0.0146 (7)	0.0161 (7)	0.0115 (7)	0.0006 (6)	0.0047 (5)	−0.0019 (6)
C2	0.0153 (7)	0.0126 (7)	0.0110 (7)	0.0019 (6)	0.0048 (5)	−0.0006 (6)
C3	0.0183 (7)	0.0149 (7)	0.0138 (7)	−0.0010 (6)	0.0049 (6)	0.0006 (6)
C4	0.0205 (8)	0.0245 (8)	0.0153 (7)	−0.0030 (7)	0.0081 (6)	−0.0011 (6)
C5	0.0171 (7)	0.0163 (7)	0.0121 (7)	0.0027 (6)	0.0054 (6)	0.0001 (6)
C6	0.0159 (7)	0.0188 (8)	0.0141 (7)	−0.0035 (6)	0.0039 (6)	−0.0015 (6)
C7	0.0246 (8)	0.0243 (8)	0.0118 (7)	−0.0063 (7)	0.0075 (6)	−0.0017 (6)
C8	0.0254 (9)	0.0310 (9)	0.0226 (8)	−0.0068 (8)	0.0127 (7)	−0.0089 (7)

### Geometric parameters (Å, °)

**Table:**

O1—C1	1.2256 (18)	C3—H3A	0.9900
O2—C5	1.2164 (18)	C3—H3B	0.9900
O3—C6	1.3974 (18)	C4—H4A	0.9800
O3—C7	1.4461 (17)	C4—H4B	0.9800
N1—C1	1.3654 (19)	C4—H4C	0.9800
N1—C5	1.3839 (19)	C6—H6A	0.9900
N1—H1	0.889 (19)	C6—H6B	0.9900
N2—C2	1.2894 (19)	C7—C8	1.509 (2)
N2—N3	1.3688 (16)	C7—H7A	0.9900
N3—C5	1.3821 (18)	C7—H7B	0.9900
N3—C6	1.4686 (19)	C8—H8A	0.9800
C1—C2	1.4832 (19)	C8—H8B	0.9800
C2—C3	1.496 (2)	C8—H8C	0.9800
C3—C4	1.536 (2)		
			
C6—O3—C7	114.18 (11)	H4A—C4—H4C	109.5
C1—N1—C5	125.26 (13)	H4B—C4—H4C	109.5
C1—N1—H1	117.7 (12)	O2—C5—N3	123.49 (14)
C5—N1—H1	117.0 (12)	O2—C5—N1	122.24 (13)
C2—N2—N3	119.17 (12)	N3—C5—N1	114.26 (13)
N2—N3—C5	124.75 (12)	O3—C6—N3	112.50 (12)
N2—N3—C6	114.37 (11)	O3—C6—H6A	109.1
C5—N3—C6	120.88 (12)	N3—C6—H6A	109.1
O1—C1—N1	122.90 (13)	O3—C6—H6B	109.1
O1—C1—C2	122.78 (13)	N3—C6—H6B	109.1
N1—C1—C2	114.33 (12)	H6A—C6—H6B	107.8
N2—C2—C1	122.02 (13)	O3—C7—C8	107.51 (12)
N2—C2—C3	119.31 (12)	O3—C7—H7A	110.2
C1—C2—C3	118.61 (13)	C8—C7—H7A	110.2
C2—C3—C4	111.76 (12)	O3—C7—H7B	110.2
C2—C3—H3A	109.3	C8—C7—H7B	110.2
C4—C3—H3A	109.3	H7A—C7—H7B	108.5
C2—C3—H3B	109.3	C7—C8—H8A	109.5
C4—C3—H3B	109.3	C7—C8—H8B	109.5
H3A—C3—H3B	107.9	H8A—C8—H8B	109.5
C3—C4—H4A	109.5	C7—C8—H8C	109.5
C3—C4—H4B	109.5	H8A—C8—H8C	109.5
H4A—C4—H4B	109.5	H8B—C8—H8C	109.5
C3—C4—H4C	109.5		
			
C2—N2—N3—C5	−2.5 (2)	C1—C2—C3—C4	74.86 (17)
C2—N2—N3—C6	178.28 (12)	N2—N3—C5—O2	−175.83 (13)
C5—N1—C1—O1	176.71 (14)	C6—N3—C5—O2	3.3 (2)
C5—N1—C1—C2	−4.0 (2)	N2—N3—C5—N1	3.3 (2)
N3—N2—C2—C1	−1.8 (2)	C6—N3—C5—N1	−177.56 (12)
N3—N2—C2—C3	175.47 (12)	C1—N1—C5—O2	179.42 (14)
O1—C1—C2—N2	−175.88 (14)	C1—N1—C5—N3	0.3 (2)
N1—C1—C2—N2	4.8 (2)	C7—O3—C6—N3	−68.72 (15)
O1—C1—C2—C3	6.9 (2)	N2—N3—C6—O3	−74.79 (15)
N1—C1—C2—C3	−172.48 (12)	C5—N3—C6—O3	106.00 (15)
N2—C2—C3—C4	−102.47 (16)	C6—O3—C7—C8	−169.27 (13)

### Hydrogen-bond geometry (Å, °)

**Table:**

*D*—H···*A*	*D*—H	H···*A*	*D*···*A*	*D*—H···*A*
N1—H1···O1^i^	0.89 (2)	1.95 (2)	2.837 (2)	174.1 (17)

Symmetry codes: (i) −*x*+3/2, *y*−1/2, −*z*+3/2.
